# MicroRNA-34a inhibits cells proliferation and invasion by downregulating Notch1 in endometrial cancer

**DOI:** 10.18632/oncotarget.22770

**Published:** 2017-11-30

**Authors:** Zhen Wang, Wei Wang, Kangrong Huang, Yueling Wang, Jing Li, Xinyuan Yang

**Affiliations:** ^1^ Department of Gynecology and Obstetrics, The First Affiliated Hospital of Xi’an Jiaotong University, Xi’an 710061, P. R. China; ^2^ Department of Anesthesiology, The First Affiliated Hospital of Xi’an Jiaotong University, Xi’an 710061, P. R. China; ^3^ Center for Translational Medicine, The First Affiliated Hospital of Xi’an Jiaotong University, Xi’an 710061, P. R. China; ^4^ Department of Gynecology and Obstetrics, Northwest Women’s and Children’s Hospital, Xi’an 710003, P. R. China

**Keywords:** microRNA-34a, Notch1, endometrial cancer, proliferation, invasion

## Abstract

MicroRNAs (miRNAs) are small non-coding RNAs composed of 18-25 nucleotides that regulate the expression of approximately 30% of human protein coding genes. Dysregulation of miRNAs plays a pivotal role in the initiation and progression of malignancies. Our study has shown that microRNA-34a (miR-34a) was upregulated in human endometrial cancer stem cells (ECSCs). However, it is unknown how miR-34a regulates endometrial cancer itself. Here, we report that miR-34a directly and functionally targeted Notch1. MiR-34a inhibited the proliferation, migration, invasion, EMT-associated phenotypes by downregulating Notch1 in endometrial cancer cells. Overexpression of miR-34a also suppressed tumor growth in nude mice. Importantly, further results suggested miR-34a was significantly downregulated in endometrial cancer tissues and negatively correlated with Notch1 expression. There was a significant association between decreased miR-34a expression and worse patient prognosis. Taken together, our results suggest that miR-34a plays tumor-suppressive roles in endometrial cancer through downregulating Notch1. Thus miR-34a could be a potential therapeutic target for prevention and treatment of endometrial cancer.

## INTRODUCTION

Endometrial cancer is the most common gynecologic cancer in developed nations. The annual incidence is projected to increase and the average age of onset has fallen [1]. Its incidence is predicted to escalate by 50-100% in 2025 with a parallel increase in associated mortality [2]. These may be due to absorption of exogenous hormone, obesity, late menopause, high pressure, etc [3]. Patients are generally treated with surgery [4], and in some cases, a combination of chemotherapy, radiotherapy, and hormone therapy [5]. The five-year survival rate in patients was only approximately 80%, while about 15-20% develop metastasis [6]. Understanding the mechanisms for the initiation and progression of endometrial cancer may provide a basis for the development of novel therapeutic strategies.

MicroRNAs (miRNAs) are a class of endogenous, short, noncoding RNAs that silence gene expression by binding to the 3′-untranslated regions (3′-UTR) of target mRNAs, inhibiting their translation and/or causing their degradation [7, 8]. The discovery of miRNA has brought new insights into the pathogenesis of various types of diseases, including cancer [9]. The crucial role of miRNA in cancer is related to their regulation of essential cellular processes and pathways [10-12]. MicroRNAs have different functions based on the tumor type in which it is found. Previous studies have identified miR-21, miR-27, miR-96, miR-128, miR-155 and miR-182 as oncogenes, and miR-17, miR-27, miR-125, miR-145, miR-205 and miR-206 as tumor suppressor genes [13-15]. Several miRNAs have been identified in normal endometrial tissues [16, 17], but the possible involvement of miRNAs expression in endometrial cancer remains unclear.

In this report, we found differential expression profiles of miRNA in endometrial cancer stem cells (ECSCs) compared with their differentiated progeny cells. Among them, miR-34a was significantly upregulated in ECSCs. This finding suggested that miR-34a may play an important role in progression and metastasis of endometrial cancer. MiR-34a is a member of the conserved miR-34 family (miR-34a, miR-34b and miR-34c) and its coding sequence differs from the other members. MiR-34a is encoded by one transcript, while miR-34b and miR-34c are encoded by another transcript allowing their diverse functions [18]. Previous studies have reported that miR-34a acts as tumor suppressor in many types of other solid tumors [19-22]. The effects of miRNAs was dependent on cell-type and exhibit diverse function through distinct pathways, as miR-34a also supported cell proliferation in HeLa and MCF-7 cells [23]. In the present study, we proved miR-34a inhibits proliferation, migration, and invasion of endometrial cancer cells. Furthermore, we verified that miR-34a acts as a tumor suppressor by downregulating Notch1 in endometrial cancer. These results indicated that the miR-34a / Notch1 axis plays a vital role in progression and invasion of endometrial cancer cells and is a potential target for prevention and treatment of endometrial cancer.

## RESULTS

### Isolation and characterization of endometrial cancer stem cells

**Figure 1:**
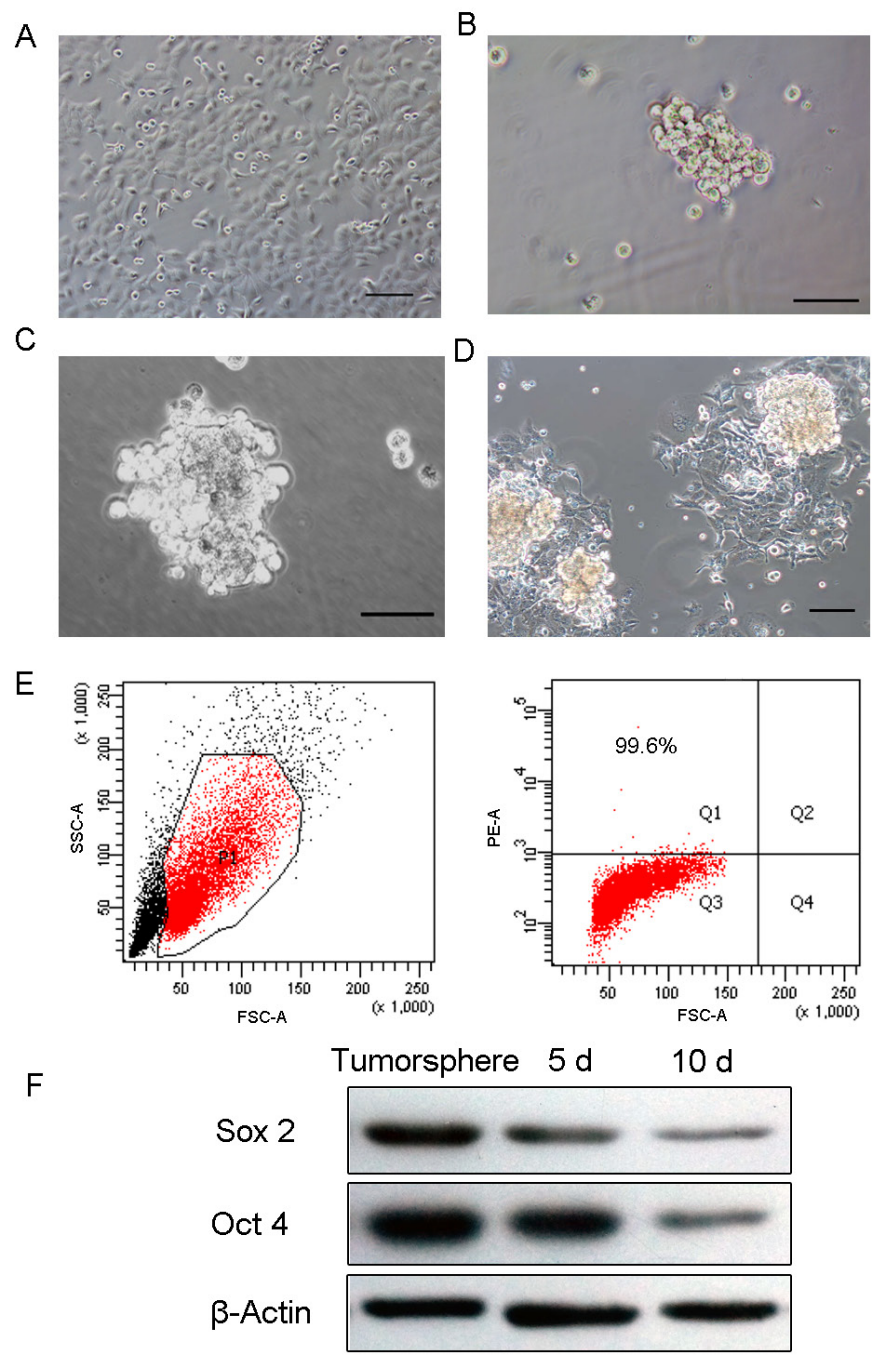
Tumorsphere formation and differentiation **(A)** HEC-1-B cells grew as an adherent monolayer. **(B)** Primary tumorspheres formed in SFM medium. **(C)** Typical tumorspheres formed in SFM at 10 days. **(D)** Tumorsphere differentiated at 3 days in DMEM/F12 medium supplemented with 10% FBS. **(E)** High-level expression of stem cell marker CD133 in tumorspheres. **(F)** Oct4 and Sox2 were significantly elevated in tumorspheres compared to 5-day and 10-day differentiated cells. (Scale bar=100 μm).

HEC-1-B cells, the typical endometrial cancer cell line, were cultured as adherent monolayers in DMEM/F12 supplemented with 10% fetal bovine serum (FBS) (Figure 1A). Part of the adherent cells may undergo cell death during the first 3 days in a serum-free environment and only a small percentage of HEC-1-B form primary spheres after 5 days of incubation in serum-free medium (SFM) (Figure 1B). Overtime, mammospheres become larger, each composed of 100∼200 cells after 14 days (Figure 1C). Using DMEM/F12 medium supplemented with 10% FBS, tumorspheres began to adhere and form uniform spindle shaped cells, typical of endometrial cancer cells (Figure 1D).

Previous studies have determined CD133, a novel 120 KDa five-transmembrane cell surface protein, as a representative ECSC surface marker [24, 25]. To identify if tumorsphere formation could enrich ECSC, we examined CD133 expression in tumorspheres cells. As determined by flow cytometric analysis, the percentage of CD133^+^ cells was 99.6% (Figure 1E). In addition, stemness-related genes such as Sox2 and Oct4 exhibited high expression in tumorspheres cells and the expression levels decreased as cells differentiated (Figure 1F).

### Characterization of miRNA expression profiles in ECSCs

**Figure 2:**
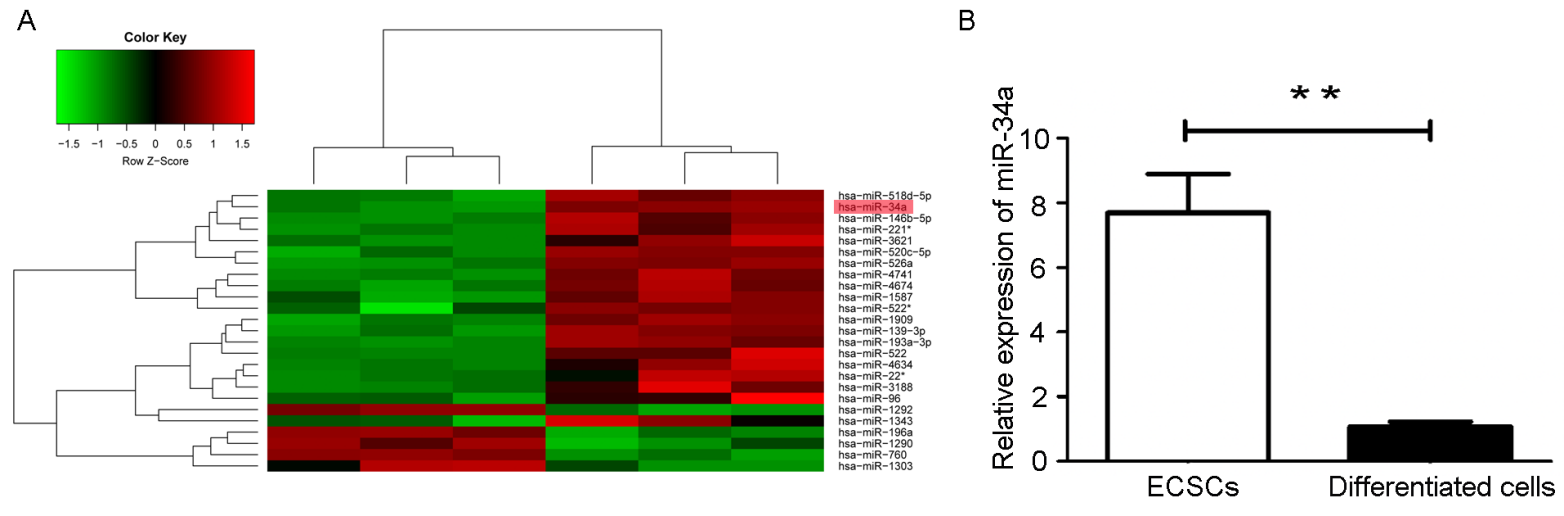
Microarray analysis of miRNAs expression level in ECSCs and its differentiated cells **(A)** Hierachical clustering analysis of miRNAs expression profiles in ECSCs and 10-day differentiated cells. Green represents low expression, red represents high expression, and black represents intermediate expression. **(B)** The expression of miR-34a was detected in ECSCs and its differentiated cells by RT-qPCR. Three independent experiments were performed. ^**^ *P* < 0.01.

To identify the miRNA expression profiles in ECSCs, miRNA microarray assays were performed and the microarray data was analyzed. Microarray analysis data shows 57 miRNAs to be differentially expressed in ECSCs compared with its differentiated progeny cells. Specifically, 11 miRNAs displayed at least a 2-fold chang in expression in ECSCs. miR-522, miR-139-3p, miR-520c-5p, miR-518d-5p, miR-146b-5p, miR-34a, miR-526a, miR-193a-3p, miR-221, miR-4674 were significantly upregulated and miR-760 was downregulated in ECSCs (Figure 2A). Among them, miR-34a was seen to be related to the self-renewal and proliferation of cancer stem cells [26-29]. RT-qPCR studies also show that miR-34a expression was upregulated in ECSCs and downregulated in differentiated cells (Figure 2B).

### miR-34a targeted Notch1

**Figure 3:**
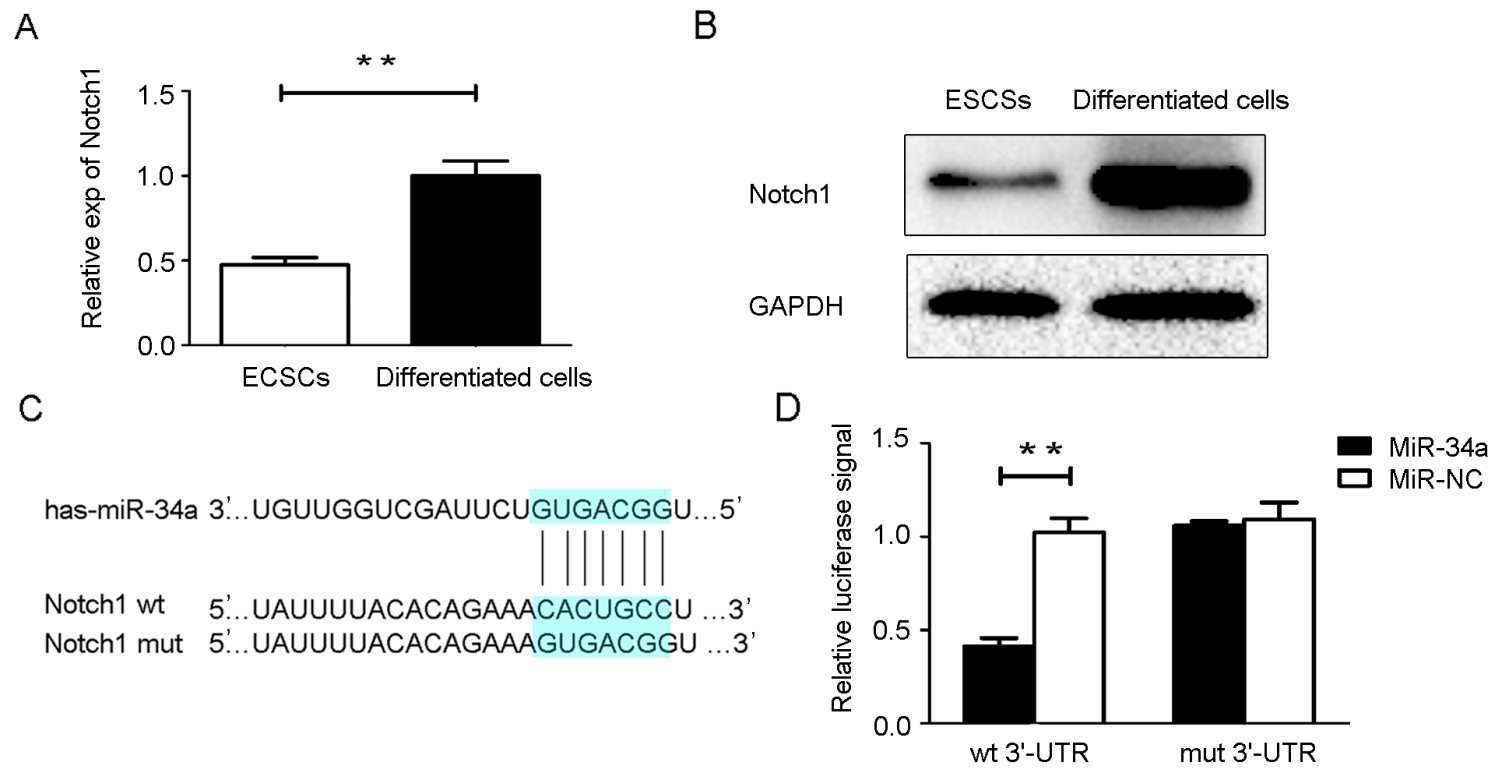
MiR-34a directly targets Notch1 **(A, B)** Notch1 mRNA and protein expression were determined in ECSCs and 10-day- differentiated cells. **(C)** The wild type (wt) and mutant (mut) binding sites for miR-34a in the 3’-UTR of the Notch1 gene. **(D)** Luciferase reporter assays. NC: non-specific negative control. Three independent experiments were performed. ^**^P < 0.01.

The combined use of bioinformatics prediction softwares (TargetScan, TarBase and miRanda) predicted target genes of miR-34a. As a result, STRT1, Protein kinase D1, Notch1, JAG1 and Prealdolase A were highly ranked in the obtained list. Among them, we chose Notch1 to further investigate because of its important roles in human endometrial carcinogenesis. We assessed Notch1 mRNA and protein expression in ECSCs and its 10-day-differentiated cells. The results show a significant decrease in both mRNA (Figure 3A) and protein levels (Figure 3B) in ECSCs compared with their differentiated cells counterparts, suggesting a putative negative correlation between miR-34a and Notch1. Notch1 has a miR-34a binding site in the 3’-UTR region (Figure 3C). Luciferase assay data shows that the miR-34a mimics inhibited the activity of the wild-type Notch1 construct when compared with a vector control, whereas no alteration was observed with the mutant Notch1 construct, suggesting miR-34a could directly regulate Notch1 gene expression at the post-transcriptional level (Figure 3D). Luciferase activity assays, RT-qPCR, and western blotting were performed to verify the relationship between miR-34a and Notch1.

**Figure 4:**
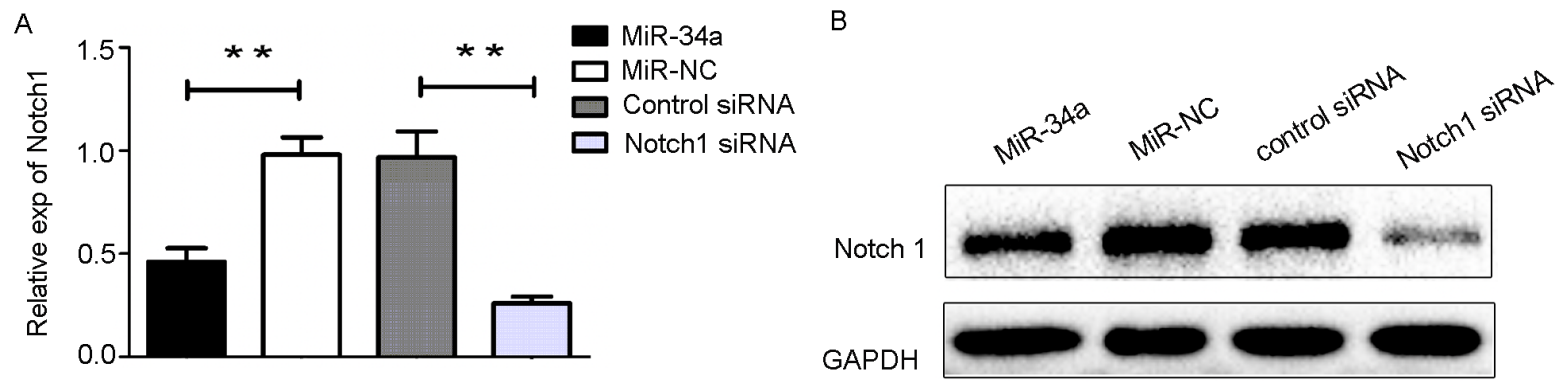
The transfection of miR3-4a mimics or Notch1 siRNA **(A)** HEC-1-B cells were transfected with miR-34a mimics, Notch1 siRNA and their corresponding controls for 48 h. The mRNA levels of Notch1 was determined by RT-qPCR. **(B)** The cells were treated as indicated above, and the protein levels of Notch1 were determined by Western blotting. NC: non-specific negative control. Three independent experiments were performed. ^**^ *P* < 0.01.

We transfected HEC-1-B cells with miR-34a mimics and its negative control for 48 h. Notch1 mRNA level was downregulated by miR-34a overexpression, consequently leading to the same protein expression profile (Figure 4A, 4B). Taken together, these results revealed that miR-34a targeted Notch1 by regulating the relevant 3’-UTR regions and repressing translation, thereby suppressing the Notch1 protein level.

### MiR-34a inhibit the cells proliferation, migration, and invasion by targeting Notch1

**Figure 5:**
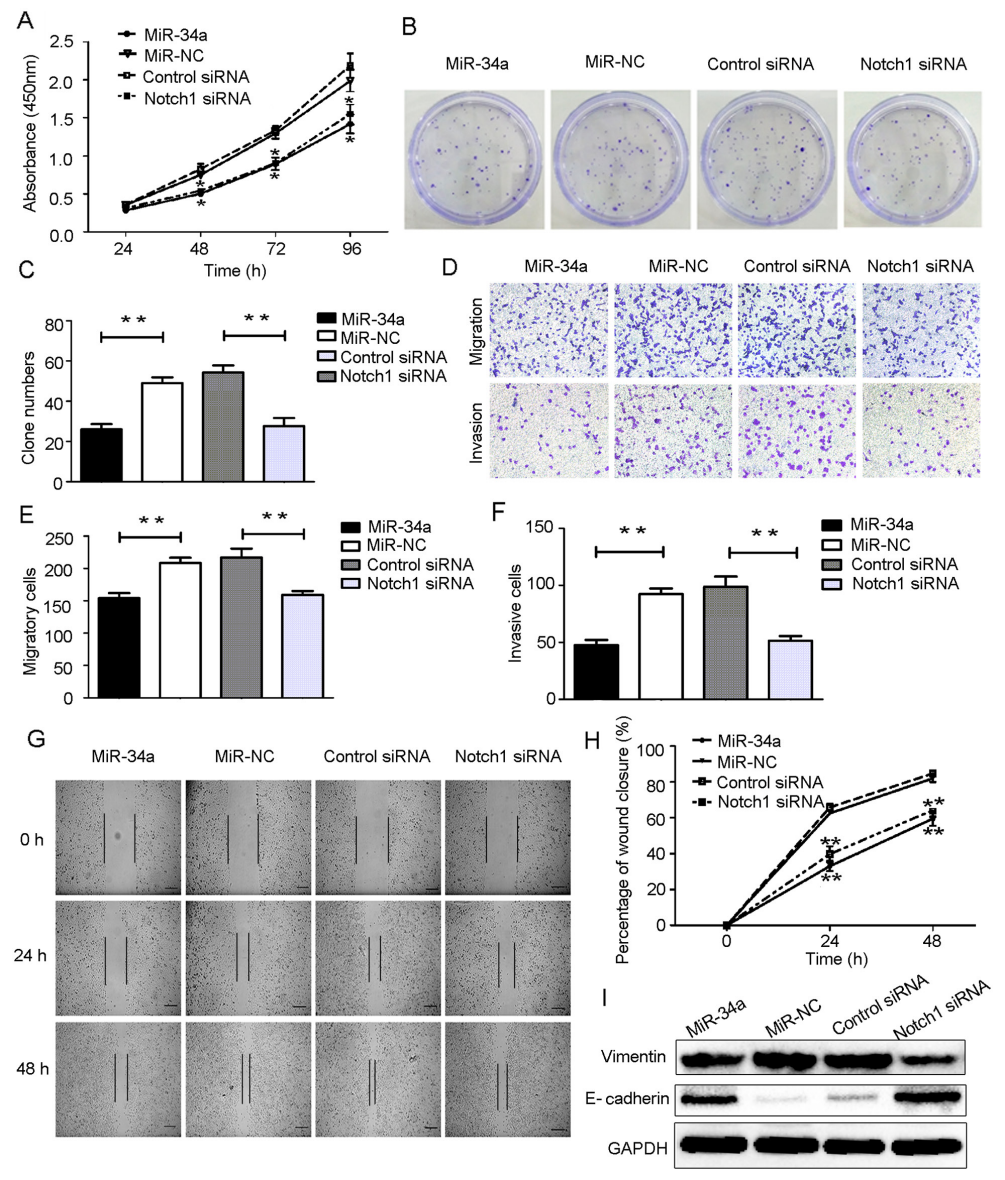
Overexpression of miR-34a suppressed HEC-1-B cells proliferation, migration, invasion and EMT by targeting Notch1 **(A)** HEC-1-B cells were transfected with miR-34a mimics, Notch1 siRNA and corresponding controls for 48 h. The cell proliferation ability was determined by CCK-8 assay. **(B, C)** Colony formation assays. **(D-F)** Cell migration and invasion were detected using Transwell assay. **(G, H)** Wound healing assay. Scale bar=100 μm. **(I)** Protein expressions of E-cadheirn and Vimentin were measured by Western blotting. NC: non-specific negative control. Three independent experiments were performed. ^*^P < 0.05, ^**^P < 0.01.

Based on our findings described above, we hypothesized that miR-34a may inhibit cell proliferation and suppress cell migration and invasion through downregulation of Notch1 expression. To test this hypothesis, HEC-1-B cells were transfected with miR-34a mimics and the corresponding control. Using small interfering RNA (siRNA), we knocked down the expression of Notch1 in HEC-1-B cells. Notch1 expression in HEC-1-B cells was also measured following transfection with siRNA using RT-qPCR and Western blot analysis (Figure 4A, 4B). As shown in Figure 5A, CCK-8 assays demonstrated that overexpression of miR-34a and knockdown of Notch1 significantly decreased cell proliferation at 48, 72 and 96 hours compared to miR-NC group or control-siRNA group. Colony formation assays were employed to evaluate a long-term impact. As expected, overexpression of miR-34a and knockdown of Notch1 contributed significantly to a decrease in clone numbers (Figure 5B, 5C). Subsequently, we evaluated cell migration and invasion by Transwell assays. The miR-34a overexpressing cells showed inhibition in both migration and invasion of the HEC-1-B cells. The knockdown of Notch1 exhibited the same effects of overexpressing miR-34a (Figure 5D, 5E, 5F). Furthermore, a wound healing assay was also performed to examine whether miR-34a/ Notch1 are involved in cell migration. After 24h and 48h, miR-34a mimics group and Notch1 siRNA group exhibited decelerated cell migration into the wounded areas compared to control group. MiR-34a mimics group and Notch1 siRNA group demonstrated a significantly larger percentage of remaining gap, while the control group showed lower remaining gap (Figure 5G, 5H).

Western blot analyses showed that overexpression of miR-34a and knockdown of Notch1 were associated with upregulation of epithelial marker E-cadherin and downregulation of mesenchymal marker Vimentin. These data suggest that overexpression of miR-34a or decreased expression of Notch1 can reduce the mesenchymal phenotype and invasive ability of HEC-1-B cells (Figure 5I).

### MiR-34a suppressed xenograft tumor formation

**Figure 6:**
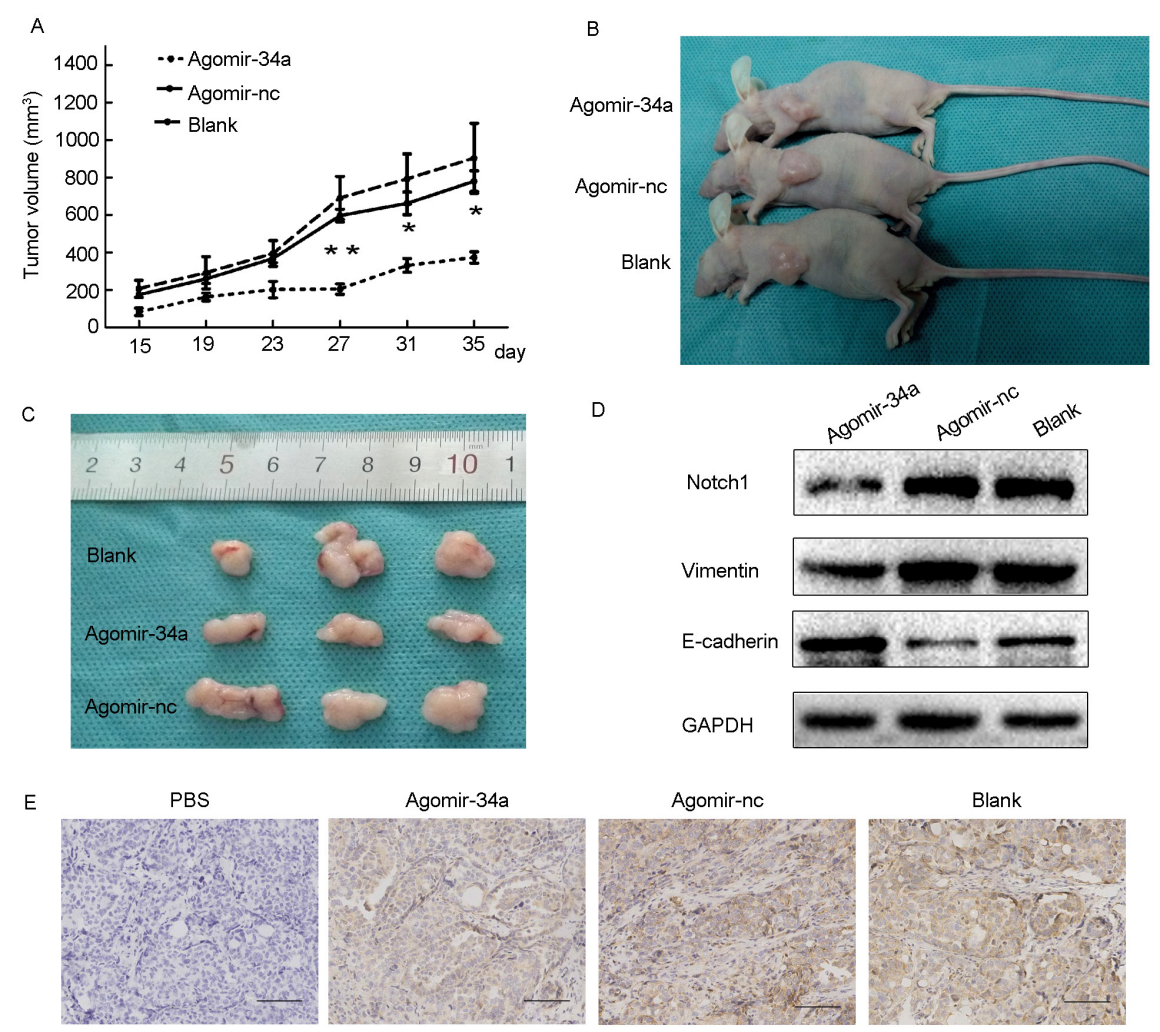
Overexpression of miR-34a inhibited tumor growth and EMT in BALB/c nude mice **(A)** Average of tumor volumes were measured after cells were injected. **(B, C)** Photograph of typical tumors grown in mice. **(D)** The protein expression of Notch1, E-cadherin and Vimentin was detected by Western blotting. **(E)** The expression of Notch1 was detected by IHC. Scale bar=190 μm. ^*^P < 0.05, ^**^P < 0.01.

To further confirm the role of miR-34a in tumor growth, HEC-1-B cells were pretreated with agomiR-34a or agomir-control for 48h *in vitro*. Cells were then subcutaneously injected into BALB/c nude mice. A significant difference in tumor volumes between the agomiR-34a group and the agomir-control group appeared from 27 days, while the difference between agomir-control group and blank group were not statistically significant (Figure 6A). As shown in Figure 6B and 6C, the volumes of tumors that grew from injected miR-34a high expressing cells were smaller than those arising from control cells, indicating miR-34a suppressed the growth of endometrial cancer cells *in vivo*. Furthermore, the results of Western blotting and IHC demonstrated that the expression of Notch1 was dramatically decreased in tumors from agomiR-34a group compared to those from agomir-control group and blank group (Figure 6D, 6E). We also found that tumors in agomiR-34a group exhibited higher E-cadherin expression and lower Vimentin expression when compared to agomir-control group (Figure 6D).

### Association between miR-34a and Notch1 in endometrial cancer tissues

**Figure 7:**
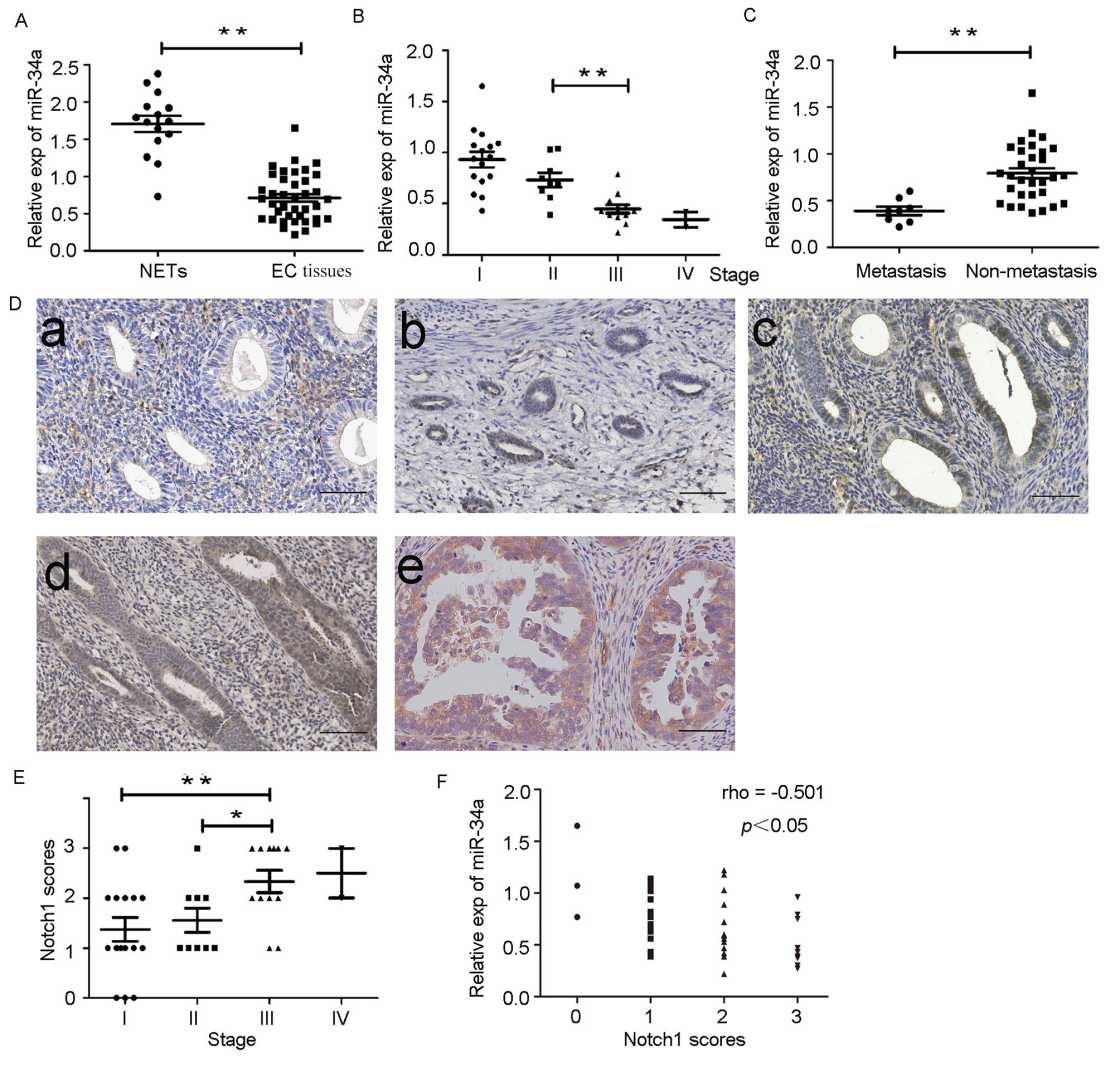
Down-regulation of miR-34a in endometrial cancer tissues is negatively correlated with expression of Notch1 **(A)** Low expression of miR-34a in endometrial cancer tissues (EC tissues) compared with normal endometrial tissues (NETs). **(B)** The expression of miR-34a in different clinical stages of endometrial cancer patients. **(C)** Low expression of miR-34a in patients with lymph node metastases. **(D)** The expression of Notch1 was detected in normal endometrial tissues and endometrial cancer tissue by IHC. a. Normal endometrial tissues samples; b. Endometrial cancer tissues samples from patients with stage I; c. Endometrial cancer tissues samples from patients with stage II; d. Endometrial cancer tissues samples from patients with stage III ; e Endometrial cancer tissues samples from patients with stage IV. (Scale bar=70μm). **(E)** The pathologic score of Notch1 expression in endometrial cancer tissues. **(F)** The expression of miR-34a was negatively correlated with Notch1 scores in endometrial cancer. ^*^P < 0.05, ^**^P < 0.01.

We determined the levels of miR-34a expression in endometrial cancer tissues (n = 39) and normal endometrial tissues (n = 15) by RT-qPCR. The average expression level of miR-34a was significantly downregulated in endometrial cancer tissues (0.71 ± 0.05) compared to the normal endometrial tissues (1.70 ± 0.11) (Figure 7A). As shown in Table 1, the level of miR-34a was reduced with progression of clinical stage. A significant difference was observed between stage II and stage III (Figure 7B). Additionally, miR-34a expression levels in patients with lymph node metastases was also lower than that in patients without lymph node metastases (Figure 7C). The results indicated that reduced expression of miR-34a may play an important role in the progression and metastasis of endometrial cancer. Notch1 expression was detected by immunohistochemistry (IHC) in the same set of samples. Notch1 was found to be overexpressed in endometrial cancer tissues compared to normal endometrial tissues (Figure 7D). Moreover, the pathologic score of Notch1 expression was induced with progression of clinical stage, but no significant difference was observed between stage III and stage IV (Figure 7E). In addition, analysis of IHC scores showed that Notch1 was negatively associated with miR-34a expression (Figure 7F).

**Table 1:** The relationship between clinicopathological parameters and miR-34a expression in endometrial cancer (n = 39)

Clinicopatholopic parameter	No. of cases	Median expression of miR-34a	*P*-value
Age, years			
>45	33	0.70 ± 0.06	>0.05
≤45	6	0.77 ± 0.12	
Lymph node metastasis			
Yes	8	0.39 ± 0.05	<0.05
No	31	0.79 ± 0.05	
TNM stage			
I	16	0.93 ± 0.07	>0.05
II	9	0.75 ± 0.08	<0.05
III	12	0.45 ± 0.04	>0.05
IV	2	0.35 ± 0.08	

## DISCUSSION

To date, although the exact mechanism of progression and metastasis in endometrial cancer remains largely unknown, it is worth noting that ECSCs have been identified in endometrial cancer tissues [30-34] and considered to be an engine of tumor evolution [35-38]. In this study, we performed enriched ECSCs by serum-free suspension cultivation. In a serum-free culture system condition, cancer stem cells initially become suspended in small clusters and detached from the parental cells. They also exhibited reduced cell–cell or cell–matrix interactions and loss of anchorage, which is necessary for the growth and sustainability of ECSCs [39]. The formed tumorspheres exhibited stem cell properties by expressing stemness-related markers. We report for the first time the miRNA expression profiles in ECSCs differ from that of their differentiated progeny cells using microarray expression assay. Interestingly, a total of 11 miRNAs, including miR-34a showed at least a 2-fold increase in ECSCs compared to its differentiated progeny cells. Therefore, we hypothesized that miR-34a plays an important role in the tumorigenesis and invasiveness of endometrial cancer. Although previous findings support a tumor suppressive role of miR-34a in human malignancies, the high expression of miR-34a is also implicated in other tumor tissues. MiR-34a may exhibit its tumor-promoting properties in certain contexts. In order to clarify the specific role of miR-34a in endometrial cancer, HEC-1-B cells were transfected with miR-34a mimics and miR-34a NC. The results show that the cell viability, number of cell-colonies, number of migratory and invasive cells in miR-34a mimics group were significantly lower than that in miR-34a NC group. Overexpression of miR-34a also remarkably reversed EMT-associated phenotypes. Thus, our data suggests that miR-34a acts as a tumor suppressor *in vitro*.

The luciferase reporter assay verified that Notch1 was a direct target gene of miR-34a. We found that the expression of Notch1 significantly decreased in both mRNA and protein levels in ECSCs. Meanwhile, the expression of Notch1 decreased in the miR-34a mimics group, suggesting that miR-34a regulates Notch1 in a negative manner. In addition, we found that knockdown of Notch1 exhibited the same effects of overexpressing miR-34a. From these two aspects, it could be well concluded that miR-34a inhibited tumor formation via downregulating Notch1.

A nude mouse model was employed to identify the *in vivo* effects of miR-34a on tumorigenesis of endometrial cancer. We observed that miR-34a overexpression significantly suppressed tumor growth, reduced the expression of Notch1, and reversed the EMT-associated phenotypes. Thus, the high expression of miR-34a also shows anti-tumor efficacy *in vivo*.

It was interesting to note that a tumor-suppressive role for miR-34a has been shown in human endometrial cancer. Our current results reveal that miR-34a is significantly downregulated in endometrial cancer tissues and negatively correlated with Notch1 expression. There was a significant association between decreased miR-34a expression and worse patient prognosis.

In conclusion, our study revealed that miR-34a inhibited cell proliferation, invasion and migration by downregulating the Notch1 gene expression level *in vitro* and *in vivo*. The findings provide a tantalizing hint that miR-34a might be a promising approach for “miRNA replacement therapy” in endometrial cancer.

## MATERIALS AND METHODS

### Cell culture

The human endometrial cancer cell line HEC-1-B was purchased from the Shanghai Institute of Cell Biology of Chinese Academy of Sciences (Shanghai, China). The adherent monolayer HEC-1-B cells were cultured in serum-supplemented medium (SSM), containing Dulbecco’s modified Eagle’s medium /F12 medium (DMEM/F12, HyClone, Logan, UT, USA), 10% FBS (Gibco Laboratories, Grand Island, NY, USA), penicillin (100 U/ml) and streptomycin (100 μg/ml). The HEC-1-B cells were incubated in a 5% CO2 humidified incubator at 37°C and the medium was changed every three days.

The parental adherent monolayer HEC-1-B cells were collected and plated in 100-mm dishes (Corning, New York, USA) at a density of 5.0×10^5^ cells. When the cells were at 70% confluence, the medium was changed with SFM, containing DMEM/F12, 2% B27 supplements, 20 ng/ml epidermal growth factor (EGF) and basic fibroblast growth factor (bFGF) (Peprotech, Rocky Hill, USA), 1% Insulin-Transferrin-Selenium (ITS, Gibco, NY, USA), 5% bovine serum albumin (BSA) (Roche, Basel, Switzerland), 1% penicillin-Streptomycin and suspended in a 6-well low attachment surface well plate (Corning, NY, USA) for the sphere formation. Fresh SFM was added every other day in place of the old media. All cells were incubated in a 5% CO2 humidified incubator at 37°C. Use 0.05% trypsine/EDTA and StemPro Accutase (Gibco, NY, USA) respectively as the cell dissociation reagent. The tumor spheres were collected using a cell screen cloth for further experiments.

To induce differentiation, the tumorspheres were seeded on 60-mm dishes and cultured in DMEM/F12 with 10% FBS. Tumorsphere cells adhered on the surface of 60-mm dishes and were cultured for 10 days for complete differentiation.

### Flow cytometry

CD133 expression in ECSCs was measured by a FACScan flow cytometer (BD Biosciences, USA). ECSCs were isolated from the HEC-1-B using suspension cultivation as noted above, respectively. Sphere cells were dissociated into single cells and resuspended at a concentration of 1.0×10^7^ in 100μL buffer. CD133 antibody and isotype control antibodies (Miltenyi Biotechnology, Bergisch Gladbach, Germany) were added and samples were mixed well and incubate at 4°C for 30 minutes. Cell pellets were resuspended in a suitable amount of buffer for analysis by flow cytometry.

### Microarray assays

Total RNA, including small non-coding miRNA, was extracted from tumorspheres and 10-day-differentiated cells using TRIzol Reagent (Invitrogen, Garlsbad, CA, USA) and treated with DnaseI. The purity of RNA was assessed by Nano-Drop 2000 (Thermo, USA), and only highly pure RNAs (1.7< A260/A280 <2.2) were used for downstream experiments. The hybridization, scanning and data analysis of miRNA chip performed by Shanghai Jikai GeneChemical Technology Co. Ltd. (Shanghai, China). Three miRNA target databases were used in the analysis of the target genes of differentially expressed miRNAs, including TargetScan, miRanda and TarBase.

### Luciferase assay

For dual luciferase assays, the 3′-UTR of Notch1 containing the predicted or mutated binding sites were amplified by RT-PCR and then subcloned into the pmiR-REPORTTM vector (RiboBio, GuangZhou, China). HEC-1-B cells were seeded into 24-well plates and then co-transfected with miR-34a mimics, miR-NC and luciferase reporter plasmids (either the wild-type or mutant 3’-UTR of the Notch1 gene plasmid) using Lipofecatamine 2000 (Invitrogen, Carlsbad, CA). After transfection for 48 hours, a luciferase assay kit (Promega, Madison, WI, USA) was used to measure the luciferase activity according to the manufacturer’s protocol. Three independent experiments were carried out.

### Total RNA extraction and quantitive PCR

Total RNA was extracted using TRIzol Reagent (Invitrogen, Garlsbad, CA, USA). The purity of RNA was assessed by Nano-Drop 2000 (Thermo, USA) and only highly pure RNAs (1.7< A260/A280 <2.2) were usable. For mRNA analysis, 1 μg of total RNA from each sample was used in the reverse transcription reaction using a Revert Aid First Strand cDNA Synthesis Kit (Fermentas, USA) and Real-time qPCR was performed with a SYBR Premix Ex Taq II (Takara, China) and a CFX-96 Real-time PCR Detection System (Bio-Rad, USA). GAPDH was used as control. For miRNA analysis, 1.5 μg of total RNA from each sample was used to sythesize complementary DNA using TaqMan reverse transcription kit (Applied Biosystems, Foster City, CA, USA) and Real-time qPCR was performed with a TaqMan MicroRNA Assay kit (Applied Biosystems, Foster City, CA, USA) and a CFX-96 Real-time PCR Detection System (Bio-Rad, USA). U6 was used as control. All the data were analyzed using the 2 ^−ΔΔCt^ method and all experiments were carried out in triplicate. The specific primers (Genomics Institute, China) were shown in Table 2.

**Table 2:** Primer list

Genes	Primer sequence (5’→3’)	Product size (bp)
Notch1	F:GCAGTTGTGCTCCTGAAGAA R:CGGGCGGCCAGAAAC	80
Vimentin	F:AAGTTTGCTGACCTCTCTGAGGCT R: CTTCCATTTCACGCATCTGGCGTT	166
E-Cadherin	F:GCTGCTCTTGCTGTTTCTTCG R: CCGCCTCCTTCTTCATCATAG	108
GAPDH	F:GCACCGTCAAGGCTGAGAAC R: TGGTGAAGACGCCAGTGGA	138

### Western blot analysis

Total protein was extracted with 1× RIPA buffer (Sigma-Aldrich St. Louis, MO, USA) and BCA-200 Protein Assay Kit (Heart, China) was applied to measure protein concentration. Protein sample (40μg) underwent electrophoresis on 10% SDS-PAGE, transferred onto PVDF membranes and blocked in 5% non-fat milk. The membranes were then incubated with primary antibodies of Notch1 (1:1000, Abcam, UK), Vimentin (1:8000, Abcam, UK), E-cadherin (1:5000, Abcam, UK), Oct 4 (1:1000, Cell Signaling Technology, MA), Sox2 (1:1000, Cell Signaling Technology, MA) overnight at 4°C, followed by incubation with horseradish peroxidse-labeled secondary antibodies. The proteins were detected using ECL reagents (Millipore, USA) by a chemiluminescence imaging system (Bio-Rad). All protein expression levels were normalized to the level of the internal standard control, GAPDH (1:5000, Abcam, UK) or β-actin (1:5000, Santa Cruz, USA).

### Cell proliferation assay

Cells were seeded at 2.0×10^3^ cells/well in 96-well plates (Corning, NY, USA) and cultured in SSM. On each of the following 24, 48, 72, and 96 h, 10 μl of the Cell Counting Kit-8(CCK8, 7-Sea Biotech, China) were added to the medium at the same time point, followed by incubating at 37°C for 2 h. Absorbance values were determined at 450nm using ELISA reader (Model 680; Bio-Rad, USA).

### Colony-forming assay

Two hundred cells in each group were seeded on 60-mm cell culture dishes and cultured for 12 days at 37°C in 5% CO2. The medium was changed every 6-7 days. Cells were fixed in methanol and stained with Giemsa solution. Only colonies containing 50 or more cells were regarded as colonies and were subsequently counted.

### Migration and invasion assays

Transwell chambers (Corning, NY, USA) placed in 24-well plates (Corning, NY, USA) were used in migration and invasion assays. For invasion assay, 4.0×10^4^ cells in 100 μl SFM were placed into the upper chamber while the lower chamber contained 600μl SSM. After incubation for 24h at 37°C in 5% CO2, cells on the upper chamber were removed using cotton swabs and migrated cells on the lower chamber were fixed with paraformaldehyde for 20 min and stained with 0.1% crystal violet for 30 min. After washing with PBS, the invasive cells were counted from images of five random fields using an inverted microscope (Olympus, Japan). For invasion assay, 1.0×10^5^ cells in 100 μl serum-free medium were added to the upper chamber with Matrigel (BD Bio-sciences, San Jose, CA, USA) coated and the incubating time was prolonged to 36 hours. Other operating steps were the same as the invasion assay.

### Wound healing assay

To test the migration capacity of cells, a wound healing assay was performed. Cells were seeded in 6-well plates and transfected with miR34a mimics, Notch1 siRNA and their corresponding controls followed by incubation at 37°C in 5% CO2 for 48h. Cells were switched to serum-free medium and an injury line was made using a 200μl sterile pipette tip. The cells were incubated for additional 48 h and photographs were acquired after 0, 24 and 48h at the same injury position of each group. The migration area was analyzed.

### Xenografted tumor model

To determine the function of miR-34a *in vivo*, agomir-34a (10μm) and agomir-nc (10μm) were purchased from Ribibio (Ribobio, GuangZhou, China). Female nude mice aged 4 to 5 weeks were randomly and averagely divided into the following three groups: Blank group (mice without any treatment); Agomir-nc group (mice were injected with agomir-nc-transfected cells); and Agomir-34a group (mice were injected with agomir-34a-transfected cells). After transfection, 1.0×10^7^ cells suspended in 150 μl PBS were injected subcutaneous into the mice in each group. Tumors were measured every 4 days using a vernier caliper and the volume of tumors were calculated according to the formula: length × width ^2^ /2. At 35 days after injection, the animals were sacrificed and the tumors were collected for subsequent experiments. The experimental protocol was approved by the Ethical Committee and the Institutional Animal Care and Use Committee of Xi’an Jiaotong University.

### Clinical samples

Tissue samples were collected from the Department of Gynecology, the First Affiliated Hospital of Xi’an Jiaotong University (Xi’an, China) and the study was approved by the local ethics committee. Informed consent was obtained from each patient. No patient had undergone any preoperative chemotherapy or radiotherapy. Samples were collected immediately after surgical resection, clinical stage and histologic classification were based on the International Federation of Gynecology and Obstetrics classification system. Histologic diagnoses were reviewed by independent pathologists.

### Immunohistochemistry

Tumors excised from animals and tissue samples from patients were fixed in formalin solution overnight, then dehydrated, paraffin-embedded and serially sectioned at 4 μm thick, followed by de-waxing through xylene cycles and rehydration through a graded series of alcohol. The antigen retrieval was carried out using 1 mM EDTA solution (PH=9.0), followed by removing of endogenous peroxidase and blocking. The sections were then incubated with Notch1 primary antibody (1:100, Abcam, Cambridge, MA) overnight at 4°C and then incubated with secondary antibody using SP-link Detection Kits (ZSGB-Bio, BeiJing, China) according to the standard protocol at room temperature. Immunoreactive proteins were detected with diaminobenzidine (DAB, ZSGB-Bio, China). Sections were counterstained with hematoxylin. Slides were evaluated by two pathologist, respectively, and scored as follows: 0, no stained cells; 1, weak staining ; 2, moderate staining; 3, intense staining.

### Statistical analysis

All data were expressed as the mean ± S.E.M from at least three independent experiments and SPSS statistical software (version 18.0; SPSS Inc., Chicago, IL) was used in data analysis. Comparison among multiple groups was analyzed by one-way analysis of variance (ANOVA), while pairwise comparison in the same group was analyzed by *t*-test. The correlation analysis was done by Pearson analysis. In all cases, differences were considered significant at *P*<0.05, or highly significant at *P*<0.01.
